# Towards the generation of polycarpic perennial rapeseeds

**DOI:** 10.1016/j.fmre.2024.11.003

**Published:** 2024-11-13

**Authors:** Dong Zhai, Xiao-Li Liu, Xiang Bao, Zhi Zhu, Yan-Fei Mao, Jia-Wei Wang

**Affiliations:** a National Key Laboratory of Plant Molecular Genetics, Chinese Academy of Sciences Center for Excellence in Molecular Plant Sciences, Institute of Plant Physiology and Ecology, Chinese Academy of Sciences, Shanghai 200032, China; b University College London, London WC1E 6BT, UK; c University of Chinese Academy of Sciences, Shanghai 200032, China; d School of Life Science and Technology, ShanghaiTech University, Shanghai 201210, China; e Key Laboratory of Plant Carbon Capture, Chinese Academy of Sciences, Shanghai 200032, China; f New Cornerstone Science Laboratory, Shanghai 200032, China

**Keywords:** Perennial crops, Brassicaceae, Rapeseed, Perenniality, *FLOWERING LOCUS C*

## Abstract

Perennial crops are increasingly recognized as a sustainable agricultural solution that provides significant advantages over annual crops, including reduced seed input and management cost, improved agronomic traits and soil health, and enhanced resource use efficiency. The current strategies for breeding perennial crops include traditional selection methods, interspecific hybridization, and *de novo* domestication using genome-editing technologies. A promising new approach involves leveraging perennial genes from wild relatives to develop these crops. In the past twenty years, researchers have successfully identified the genes responsible for polycarpic flowering behaviors in the Brassicaceae family. In this Perspective, we outline a roadmap for generating perennial *Brassica napus* (rapeseed) based on these findings. We believe that further investigation into the genetic mechanisms underlying perennial syndrome is crucial for enhancing the environmental benefits of perennial crops, which will ultimately support sustainable agricultural practices.

Compared to traditional annual crops, perennial crops offer numerous advantages, including reduced seed input, improved restoration and protection of the soil's tillage layer, as well as higher water and nutrient use efficiency [1,2]. While various breeding strategies have been employed to develop perennial crops, including traditional selection, interspecific hybridization, and *de novo* domestication through the implementation of genome-editing technologies [1,[3], [4], [5], [6]], exploiting perennial genes from wild relatives is another promising approach for developing perennial crops.

The Brassicaceae family includes many economically and nutritionally important crops. By studying *Arabis alpina, Cardamine flexuosa, Crucihimalaya himalaica*, and *Erysimum nevadense* as model species, researchers have successfully cloned the key genes that control perennial flowering behavior within the Brassicaceae family [[7], [8], [9]]. Specifically, the transition from perennial to biennial and annual flowering behavior is influenced by the additive effects of three closely-related MADS-box genes: *FLOWERING LOCUS C* (*FLC*), *FLOWERING LOCUS M* (*FLM*), and *MADS AFFECTING FLOWERING* (*MAF*). The variation in expression patterns and functional strength of these genes, along with their different combinations, allows plants to adopt a wide range of life history strategies to adapt to changing growth environments. Importantly, it has been demonstrated that annual Brassicaceae plants have the potential to become perennial; by introducing a perennial-type *FLC* gene, it is possible to convert annual Brassicaceae plants into perennial flowering plants [7].

While these findings provide a foundation for developing a variety of perennial Brassicaceae crops, achieving the perennialization of *Brassica napus* (rapeseed) poses several challenges. One significant hurdle is the strict regulations surrounding genetically modified crops in many countries, necessitating the use of genome editing technologies to achieve transgene-free perennialization in rapeseed. To accomplish this, we must develop highly efficient tools for regeneration, base editing, targeted insertion, and DNA fragment replacement to enable precise genome modifications in rapeseed (Fig. 1a). Given the close relationship between rapeseed and the model plant *Arabidopsis thaliana* - both belonging to the Brassicaceae family-, we can leverage existing technologies developed for *Arabidopsis* in our research.

**Fig. 1:**
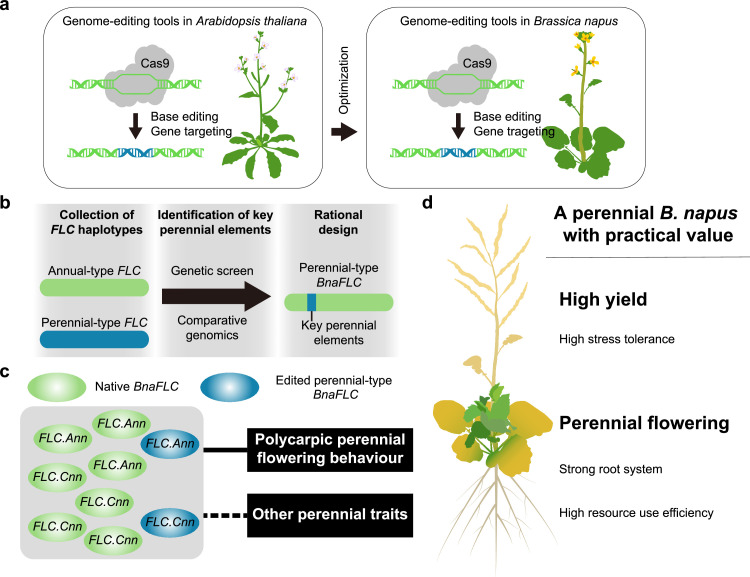
**A roadmap for generating perennial plants of *Brassica napus* (rapeseed)**. (a) Adaptation and implementation of genome editing tools developed for *A. thaliana* to use in editing *B. napus*. (b) Rational design of a perennial-type *FLC* gene using a functional genomics approach that involves three different steps: 1) collecting *FLC* haplotypes (annual- or perennial-type) from various wild species within the Brassicaceae family; 2) identifying key cis-elements determining perenniality; and 3) employing genome editing techniques (a) to convert the annual-type *FLC* gene into a perennial *B. napus* plant variant. (c) The ideal strategy to achieve a robust and viable polycarpic perennial variant of *B. napus*. This can be achieved by either converting the main effective (i.e., dominant) annual-type *BnaFLC* gene into its perennial form; or by instead increasing the number of edited perennial-type *BnaFLC* genes in the genome. The solid line denotes a clear association between the *FLC* gene and polycarpic flowering behavior, while the dashed line indicates a potential role of this gene across other perennial traits. *FLC.Ann* and *FLC.Cnn* refer to the presence of the *FLC* gene in the A and C *B. napus* subgenomes, respectively. (d) The ideal phenotype for achieving a polycarpic perennial rapeseed. While perennial flowering behavior and high-yield are the two traits warranting the highest research attention, perenniality is a syndrome composed of many other interacting traits, such as stress resistance, root anatomy, and resource use efficiency. Green and yellow colors represent newly-formed vegetative shoots after vernalization and dead plant parts, respectively.

Building on this work, our next step will be to replace the endogenous annual-type *FLC* gene in rapeseed with a perennial variant. Preliminary studies have shown that the key differences between perennial- and annual-type *FLC* genes primarily involve their expression patterns [1]. In perennial Brassicaceae species, the perennial-type *FLC* gene is only transiently silenced during vernalization. However, it is important to acknowledge that the *FLC* locus is relatively large, typically spanning from 10 to 20 kb in Brassicaceae plants, making it impractical to replace the entire locus. To address this, it will be necessary to conduct molecular mechanism studies using existing perennial model plants to identify the key DNA sequences responsible for the seasonal oscillation in the expression patterns of the *FLC* gene (Fig. 1b). Once these precise DNA sequences are identified, it will be possible to proceed with the targeted DNA replacement process. There are two possible approaches to consider, namely Base Editing Techniques and Gene Targeting Techniques (Fig. 1a).

In contrast, if the differences between the perennial and annual *FLC* types are more substantial, we need to consider implementing large fragment DNA replacement strategies to modify the *FLC* locus. For this type of replacement in *Arabidopsis*, potential methods include using geminivirus-mediated gene targeting, *Agrobacterium* T-DNA-mediated gene targeting, and PEG-mediated protoplast transfection and regeneration strategies.

It is important to note that cultivated rapeseed varieties are allopolyploids, and according to published genomic sequences and previous studies, their genomes contain a total of nine *FLC* genes [10]. Therefore, it is crucial to determine which specific *FLC* gene(s) to edit. To address this, it will be necessary to conduct a systematic study and analysis of these *FLC* genes to identify the most suitable targets for editing (Fig. 1c). Furthermore, our research on *C. himalaica* and *E. nevadense* has shown that the robustness of the perennial growth habit is closely linked to the dosage of the *FLC* gene; only under high dosage conditions do plants exhibit a stable perennial phenotype [7]. As a result, it is possible that it will be necessary to edit multiple *FLC* loci in rapeseed to achieve the desired perennial characteristics.

Upon completion of the aforementioned tasks, it will be possible to achieve a preliminary perennialization of rapeseed. However, it is important to recognize that perenniality is a complex syndrome [11,12]. Perennial flowering behavior is likely just a prerequisite for the perennialization of Brassicaceae plants [7], and it may only ensure the survival of rapeseed plants for a few years under optimal conditions, such as in a greenhouse. Therefore, to enable successful field applications, we must further investigate the key genes that determine other perennial traits such as root anatomy, resource use efficiency, regulation of source-sink dynamics, and long-term tolerance to drought, high temperatures, and pathogens [11,12] (Fig. 1d).

Lastly, an important point that should not be overlooked is the balance between crop yield and perenniality. Experimental evidence suggests that perennial plants often allocate resources to ensure their survival, potentially at the expense of seed production [12]. Currently, it remains unclear whether introducing perennial-type *FLC*-like genes will affect the number of siliques and seeds in rapeseed. Therefore, it is crucial to conduct research on existing perennial model plants, in particular focusing on whether perennial flowering behavior impacts seed set in Brassicaceae species. If it is found that perennial flowering behavior does indeed affect seed production, it will be important to investigate the associated molecular mechanisms to identify the key genes that link perennial flowering behavior and seed set. With this understanding, it will be possible to use genome editing techniques to decouple these two traits, aiming to develop rapeseed varieties that maintain high yields while exhibiting perenniality (Fig. 1d).

## Declaration of competing interest

The authors declare that they have no conflicts of interest in this work.
